# Identification of the ceRNA networks in α-MSH-induced melanogenesis of melanocytes

**DOI:** 10.18632/aging.202320

**Published:** 2020-12-14

**Authors:** Ling Jiang, Jinhua Huang, Yibo Hu, Li Lei, Yujie Ouyang, Yan Long, Hui Li, Si Li, Lun Yang, Yan Yang, Lihua Huang, Hong Xiang, Rong Xiao, Jing Chen, Qinghai Zeng

**Affiliations:** 1 Department of Dermatology, Third Xiangya Hospital, Central South University, Changsha 410013, Hunan, China; 2 Central Laboratory, Third Xiangya Hospital, Central South University, Changsha 410013, Hunan, China; 3 Department of Dermatology, Second Xiangya Hospital, Central South University, Hunan Key Laboratory of Medical Epigenetics, Changsha 410011, Hunan, China

**Keywords:** ceRNA, α-MSH, melanogenesis, ENST00000606533, circ_0091223

## Abstract

α-MSH is known for melanogenesis stimulation, and ceRNA is a new method involved in physiological regulation. However, whether ceRNA participates in α-MSH-induced melanogenesis remains unknown. We used ceRNA array to detect the expression profiles of lncRNAs, circRNAs, and mRNAs in melanocytes after α-MSH treatment. Moreover, the melanogenesis-related ceRNA regulatory networks were screened and validated. The expression profile analysis showed that 20 lncRNAs and 49 circRNAs changed five-fold after α-MSH treatment, while 933 mRNAs changed two-fold. Based on differentially expressed genes, GO and KEGG analysis were conducted and revealed that 14 genes were enriched in melanogenesis. Then, multiple lncRNA or circRNA-miRNA-mRNA ceRNA networks and lncRNA/circRNA-miRNA-mRNA quaternary ceRNA networks were identified. Thereinto, ENST00000606533, circ_0091223, and TYR expression were upregulated in α-MSH-treated melanocytes, while their complementary miR-1291 was decreased. Dual-luciferase reporter assay further verified that ENST00000606533 and circ_0091223 could bind to miR-1291. ENST00000606533 and circ_0091223 siRNAs decreased circ_0091223, ENST00000606533, and TYR expression, but increased miR-1291 expression. Conversely, miR-1291 mimics inhibited ENST00000606533, circ_0091223, and TYR expression. Moreover, miR-1291 inhibitor could reverse the inhibitory effect of the two siRNAs on TYR expression. Hence, the “ENST00000606533/circ_0091223-miR-1291-TYR” ceRNA network is involved in α-MSH-induced melanogenesis, and ceRNA networks may be potential therapeutic targets for skin pigmentation disorders.

## INTRODUCTION

Melanogenesis is a complex process, as melanin is produced in melanocytes and subsequently transferred to adjacent keratinocytes. This process is regulated by both intrinsic and extrinsic factors such as ultraviolet radiation (UVR), autocrine action, paracrine action, inflammatory, neuronal cells, and drugs. Among them, α-Melanocyte Stimulating Hormone (α-MSH) is one of the most important intrinsic factors in stimulating melanogenesis [1, 2]. When binding to melanocortin 1 receptor (MC1R), α-MSH can activate the cyclic adenosine monophosphate (cAMP)/protein kinase A (PKA)/cAMP response element-binding protein (CREB) pathway, which further promotes the expression of microphthalmia-associated transcription factor (MITF) [3, 4]. MITF can powerfully stimulate tyrosinase (TYR) expression and upregulate melanin biosynthesis. Melanogenesis is a multistage enzymatic reaction process that is regulated by various enzymes, while TYR is the key rate-limiting enzyme, the expression and activity of TYR significantly affect melanogenesis in melanocytes [5].

Recent studies have verified that less than 2% of the total genome contains protein-coding genes, but non-coding genes exist in most of the human transcriptome [6]. Non-coding RNAs (ncRNAs) show higher tissue specificity when compared to protein-coding mRNAs [7, 8], thus, functionalizing the mechanism and role of non-coding RNA will undoubtedly lead to further insight into basic physiology and disease progression. Long non-coding RNA (lncRNA), circular RNA (circRNA), and microRNA (miRNA) are functional members of ncRNAs, which are involved in the regulation of a variety of biological behaviors. miRNA can inhibit the translation of target genes by binding to the microRNA response elements (MREs) of mRNAs and initiate their degradation. Recent studies have found that MREs also exist on lncRNAs and circRNAs [9–11]. It is likely that miRNAs can bind to multiple types of RNA. Hence, different types of RNA can bind to the same miRNA through the same MREs, forming lncRNA-miRNA-mRNA or circRNA-miRNA-mRNA competitive endogenous RNA (ceRNA) regulation networks to regulate the physiopathological processes. As reported, lncRNA ABHD11-AS1 functions as a ceRNA to regulate papillary thyroid cancer progression by miR-199a-5p/SLC1A5 axis [12]. Similarly, circRNA-5692 inhibits the progression of hepatocellular carcinoma by sponging miR-328-5p to enhance DAB2IP expression [13].

For now, lncRNA H19, Urothelial Cancer Associated 1 (UCA1) has been reported to regulate melanogenesis in melanocytes [14, 15]. However, there is no report about the effect of circRNAs on melanogenesis. Furthermore, the role of ceRNA networks in α-MSH-induced melanogenesis has not been reported. Thus, we used ceRNA array to investigate the differential expression profiles of lncRNAs, circRNAs, and mRNAs in melanocytes with or without α-MSH treatment. Then, bioinformatics analysis was performed to screen ternary and quaternary ceRNA networks that might play a regulatory role in α-MSH-induced melanogenesis. Finally, we verfied that ENST00000606533, circ_0091223, and TYR mRNA could form a ceRNA network linked by miR-1291, it is suggested that ENST00000606533, circ_0091223, and miR-1291 may be novel targets in the treatment of skin pigmentation disorder.

## RESULTS

### Differentially expressed lncRNAs, circRNAs, and mRNAs in α-MSH-treated melanocytes

**Figure 1:**
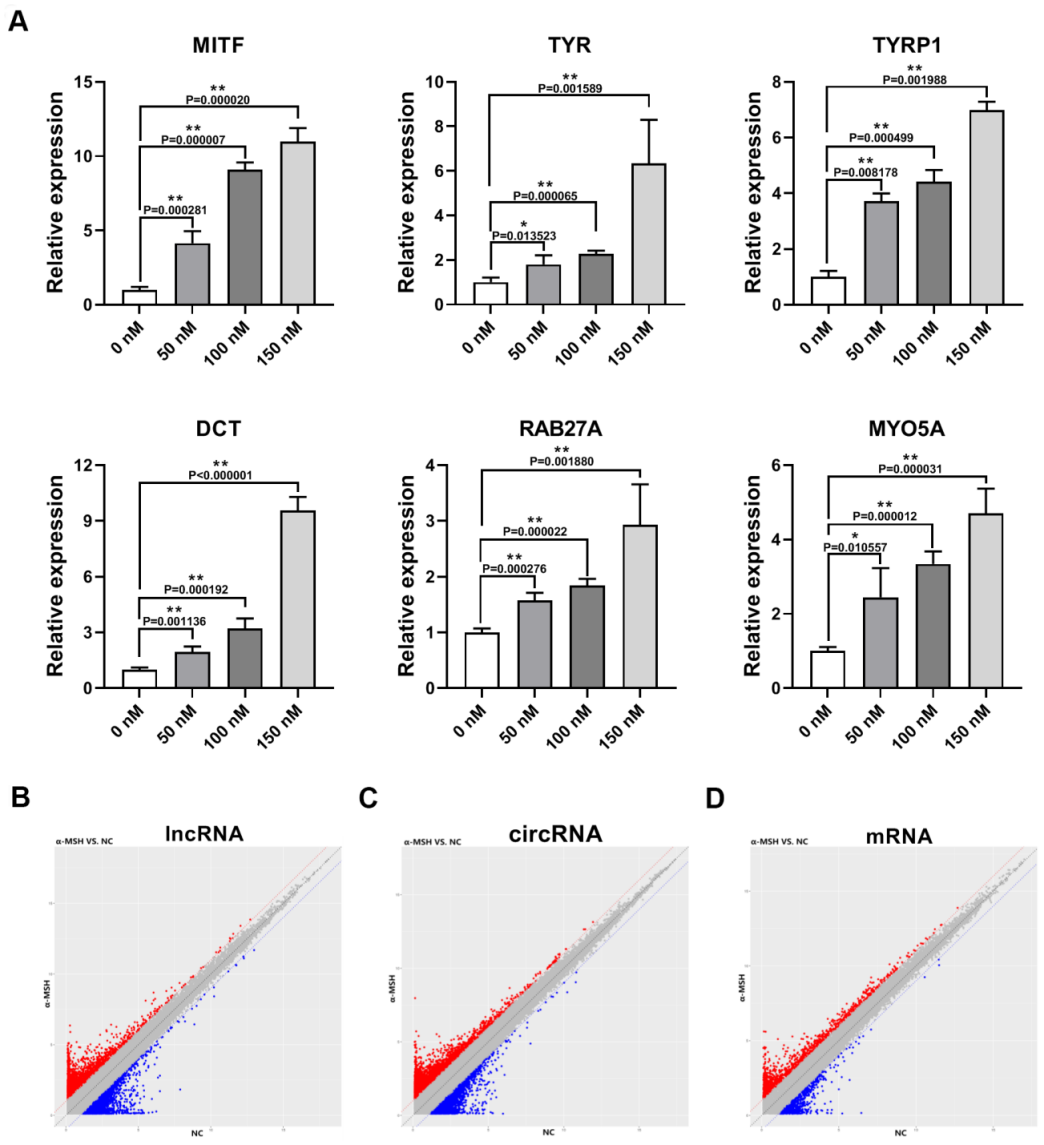
**Differentially expressed lncRNAs, circRNAs, and mRNAs in α-MSH-treated melanocytes.** (**A**) qRT-PCR was used to detect the expression of melanogenesis related genes (MITF, TYR, TYRP1, DCT, RAB27A, and MYO5A) in α-MSH-induced melanocytes. (**B**–**D**) Scatter plot of normalized expression levels for differentially expressed lncRNAs, circRNAs, and mRNAs, respectively. Red dots: upregulated lncRNAs, circRNAs, and mRNAs; Blue dots : downregulated lncRNAs, circRNAs, and mRNAs.

To induce the melanogenesis in human melanocytes, we treated human skin primary melanocytes (MCs) with different concentrations of α-MSH (0, 50, 100, 150 nM). The qRT-PCR results showed that 150 nM α-MSH increased the expression of melanogenesis-related genes [16, 17] (MITF, TYR, Tyrosinase Related Protein 1 (TYRP1), Dopachrome Tautomerase (DCT), RAB27A (Member RAS Oncogene Family), and Myosin VA (MYO5A), Figure 1A) most significantly, so we chose 150 nM α-MSH for the subsequent experiments. Then, ceRNA microarray analysis was performed to identify the differentially expressed lncRNAs, circRNAs, and mRNAs between normal control (NC, 0 nM α-MSH) and 150 nM α-MSH treated MCs. We found that 2026 lncRNAs, 3256 circRNAs, and 933 mRNAs changed by more than two-fold (Figure 1B–1D). Specifically, 11 lncRNAs were upregulated and 9 lncRNAs were downregulated by 5-fold (normalized signal value > 2) in α-MSH-treated MCs, respectively (Table 1). Furthermore, 27 circRNAs were upregulated and 22 circRNAs were downregulated by 5-fold (normalized signal value > 1.5) in α-MSH-treated MCs, respectively (Table 2). Besides, GO and KEGG pathway enrichment analysis was performed to reveal the possible involvement of significant differentially expressed mRNAs in the α-MSH-treated MCs (Supplementary Figure 1). The melanosome/GO (TYR, ATP6V1B2, ERP29, CCT4, RAN, HSPA8, RAB38, ANXA2, RPN1, SEC22B, TYROBP, and ATP1B3), and melanogenesis/pathway (EDNRB, GNAO1, FZD7, POMC, TYR, CTNNB1, FZD4, CALM2, and WNT3) indicated that these genes were significantly enriched in melanogenesis. Combined with other studies [18–20], we selected the upregulated mRNAs (TYR, SOX6, CTNNB1, IL6, FZD4, POMC, WNT3, CALM2, FZD7, MAPK11, RAB38) and the downregulated mRNAs (EDNRB, GNAO1, TYROBP) which were involved in α-MSH-induced melanogenesis (Table 3).

**Table 1:** Greater than 5-fold differentially expressed lncRNAs (normalized signal value > 2).

**Upregulated lncRNAs**			**Downregulated lncRNAs**	
**LncRNA**	**Fold change (α-MSH/NC)**		**LncRNA**	**Fold change (α-MSH/NC)**
ENST00000532071	10.10		NR_104161	0.19
NR_040100	9.40		ENST00000414633	0.19
ENST00000316807	8.73		ENST00000599817	0.18
ENST00000606533	6.86		lnc-ELAVL2-1:2	0.17
ENST00000474979	6.59		lnc-RBMY1J-6:1	0.16
lnc-CYP4F22-1:1	6.30		lnc-KIF20A-1:1	0.14
ENST00000587528	6.23		lnc-TRIM37-1:2	0.17
NR_031650	6.14		NR_104003	0.08
lnc-LNPEP-2:1	6.02		lnc-BTBD19-1:1	0.08
ENST00000530955	5.40			
ENST00000533322	5.04			

**Table 2:** Greater than 5-fold differentially expressed circRNAs (normalized signal value > 1.5).

**Upregulated circRNAs**			**Downregulated circRNAs**	
**circRNA**	**Fold change α-MSH/NC)**		**circRNA**	**Fold change (α-MSH/NC)**
circ_0031794	15.47		circ_0015226	0.20
circ_0035909	12.34		circ_0007726	0.19
circ_0074400	8.74		circ_0050401	0.19
circ_0066944	8.64		circ_0004126	0.19
circ_0030056	7.56		circ_0058630	0.18
circ_0054509	7.39		circ_0083902	0.17
circ_0015211	7.37		circ_0088002	0.17
circ_0054701	6.47		circ_0079872	0.17
circ_0068095	6.40		circ_0068584	0.17
circ_0037543	6.27		circ_0001808	0.15
circ_0040155	6.15		circ_0021740	0.15
circ_0084506	5.95		circ_0073019	0.15
circ_0002626	5.87		circ_0008626	0.15
circ_0083993	5.72		circ_0031728	0.14
circ_0025748	5.70		circ_0053546	0.14
circ_0069815	5.69		circ_0014138	0.13
circ_0053692	5.52		circ_0055832	0.13
circ_0067366	5.49		circ_0047279	0.12
circ_0059675	5.45		circ_0077787	0.10
circ_0091223	5.41		circ_0072730	0.10
circ_0031463	5.41		circ_0073464	0.09
circ_0053508	5.40		circ_0030604	0.07
circ_0020240	5.32			
circ_0074258	5.15			
circ_0058701	5.13			
circ_0002563_T_L	5.06			
circ_0026068	5.05			

### Identification of the lncRNA-miRNA-mRNA and circRNA-miRNA-mRNA ceRNA network in α-MSH-induced melanogenesis

**Figure 2:**
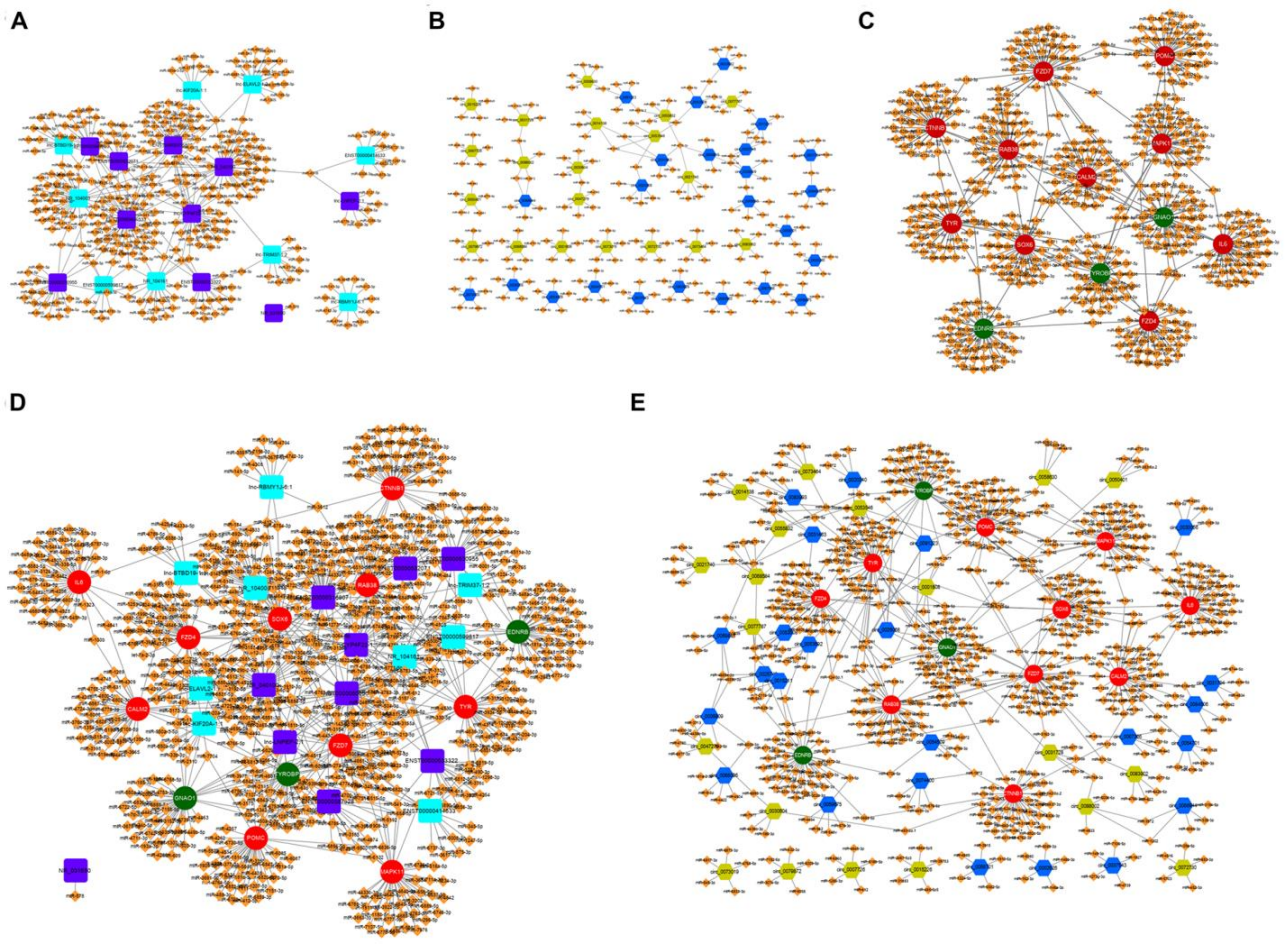
**The lncRNA-miRNA-mRNA and circRNA-miRNA-mRNA ceRNA network in α-MSH-treated melanocytes.** (**A**) Potential miRNA targets (Tot Score ≥160 or top ten Tot Score) of the differentially expressed lncRNAs with 5-fold change. (**B**) Potential miRNA targets (Top five high-binding sites) of the differentially expressed circRNAs with 5-fold change. (**C**) Potential miRNA targets (top 40 predicted miRNAs based on the score) of the differentially expressed mRNAs related to melanogenesis. (**D**, **E**) The lncRNA-miRNA-mRNA and circRNA-miRNA-mRNA ceRNA network involved in melanogenesis. Purple square: upregulated lncRNAs; Blue square: downregulated lncRNAs; Blue hexagon: upregulated circRNAs; Green six square: downregulated circRNAs; Red circle: upregulated mRNAs; Green circle: downregulated mRNAs; Orange diamond: miRNAs.

The miRanda database was used to predict the potential targeted miRNAs of the screened differentially expressed lncRNAs and circRNAs with more than 5-fold change. TargetScan databases were used to predict potential target miRNAs of differentially expressed mRNAs focused on melanogenesis. Then, predicted miRNAs of lncRNAs were screened by the condition of Tot Score ≧160, while the numbers of predicted miRNAs of lnc-BTBD19-1:1, ENST00000414633, lnc-LNPEP-2:1, lnc-RBMY1J-6:1, lnc-TRIM37-1:2, NR_031650 were few, so the top ten predicted miRNAs of these lncRNAs were screened (Figure 2A and Supplementary Table 3); the top five high-binding sites miRNAs of circRNAs were selected (Figure 2B, Supplementary Table 4); and the top 40 predicted miRNAs of mRNAs were chosen based on the score (Figure 2C and Supplementary Table 5). Furthermore, the ceRNA network of lncRNA-miRNA-mRNA (Figure 2D and Supplementary Table 6) and circRNA-miRNA-mRNA (Figure 2E and Supplementary Table 7) were constructed by Cyto Vision 3.7.0 software.

**Table 3:** Identification of differentially expressed mRNAs related to melanogenesis by GO and KEGG pathway analysis.

**Gene**	**Fold change (α-MSH/NC)**	**Gene**	**Fold change (α-MSH/NC)**
SOX6	10.16	EDNRB	0.45
CTNNB1	3.53	GNAO1	0.33
IL6	2.73	TYROBP	0.14
FZD4	2.72		
WNT3	2.47		
CALM2	2.43		
FZD7	2.32		
MAPK11	2.24		
TYR	2.18		
POMC	2.06		
RAB38	2.04		

### Identification of the quaternary ceRNA network in α-MSH-treated melanocytes

**Figure 3:**
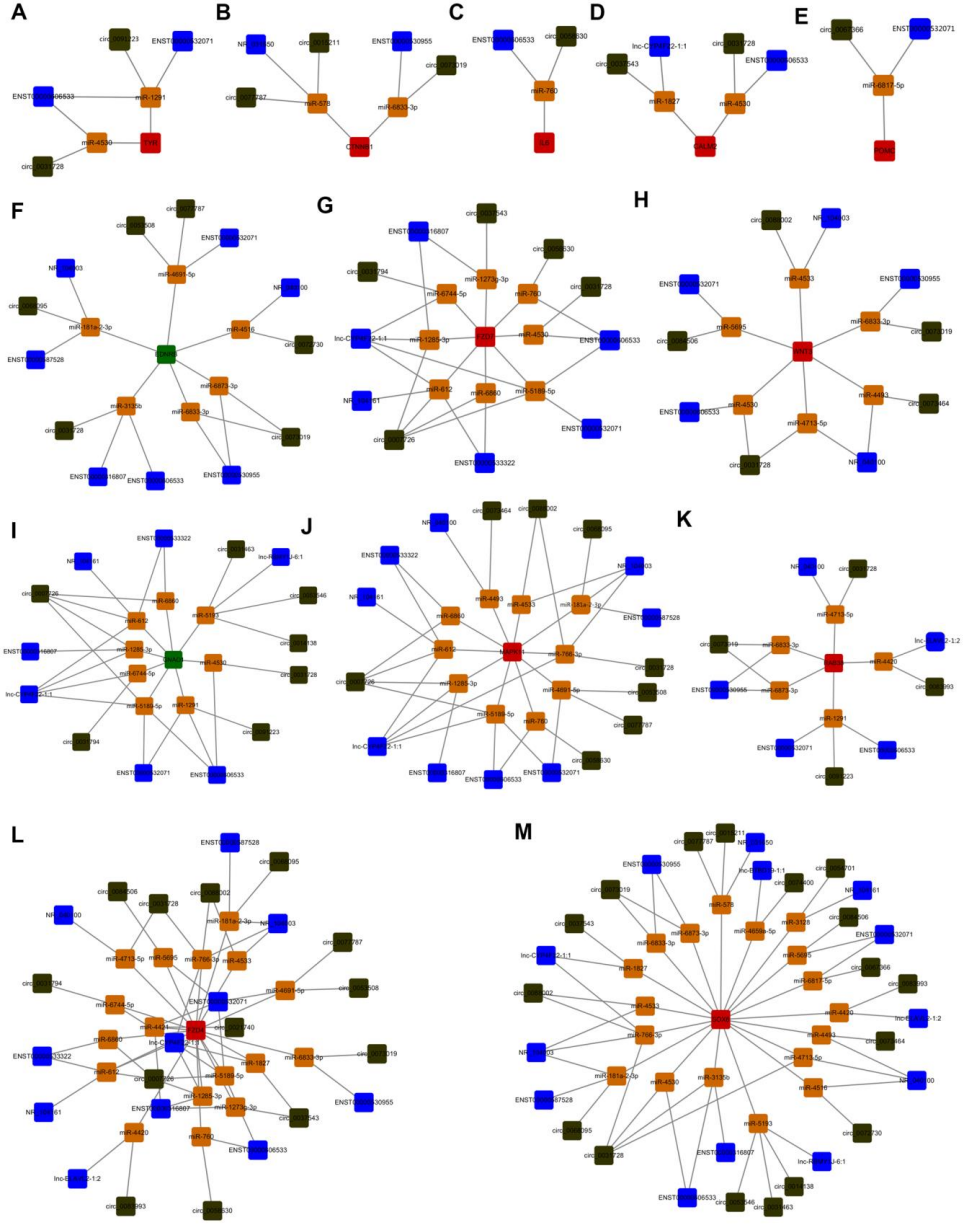
**Identification of the quaternary ceRNA network in α-MSH-treated melanocytes.** (**A**–**M**) Cyto Vision 3.7.0 was used to construct the quaternary ceRNA network focused on screened melanogenesis related mRNAs (TYR, CTNNB1, IL6, CALM2, POMC, EDNRB, FZD7, WNT3, GNAO1, MAPK11, RAB38, FZD4, SOX6). Blue square: lncRNAs; Dark green square: circRNAs; Red square: upregulated mRNAs; Green square: downregulated mRNAs; Orange square: miRNAs.

Since lncRNAs, circRNAs, and mRNAs can interact with miRNAs through the same MREs, there may exist quaternary relative network among them. Venn diagram analysis was used to screen the potential quaternary ceRNA network among the above screened lncRNAs (the corresponding 303 predicted miRNAs), circRNAs (the corresponding 192 predicted miRNAs) and melanogenesis-related mRNAs (the whole predicted miRNAs) by binding the same miRNAs (Supplementary Figure 2). Because the screened lncRNAs, circRNAs and TYROBP did not bind to the same miRNA, it did not included in the Supplementary Figures 2 and 3. Using Cyto Vision 3.7.0 for cross-linking and quaternary ceRNA network construction revealed that lncRNAs (ENST00000606533 and ENST00000532071), circ_0091223 and TYR mRNA was supposed to competitively bound with miR-1291; ENST00000606533, circ_0031728, and TYR mRNA was supposed to competitively bound with miR-4530 (Figure 3A). Other melanogenesis related mRNAs (CTNNB1, IL6, CALM2, POMC, EDNRB, FZD7, WNT3, GNAO1, MAPK11, RAB38, FZD4, and SOX6) involved in the quaternary ceRNA networks with miRNAs and above screened lncRNAs, circRNAs were presented in Figure 3B–3M.

### α-MSH treatment induced ENST00000606533 and circ_0091223 expression but inhibited miR-1291 expression

**Figure 4:**
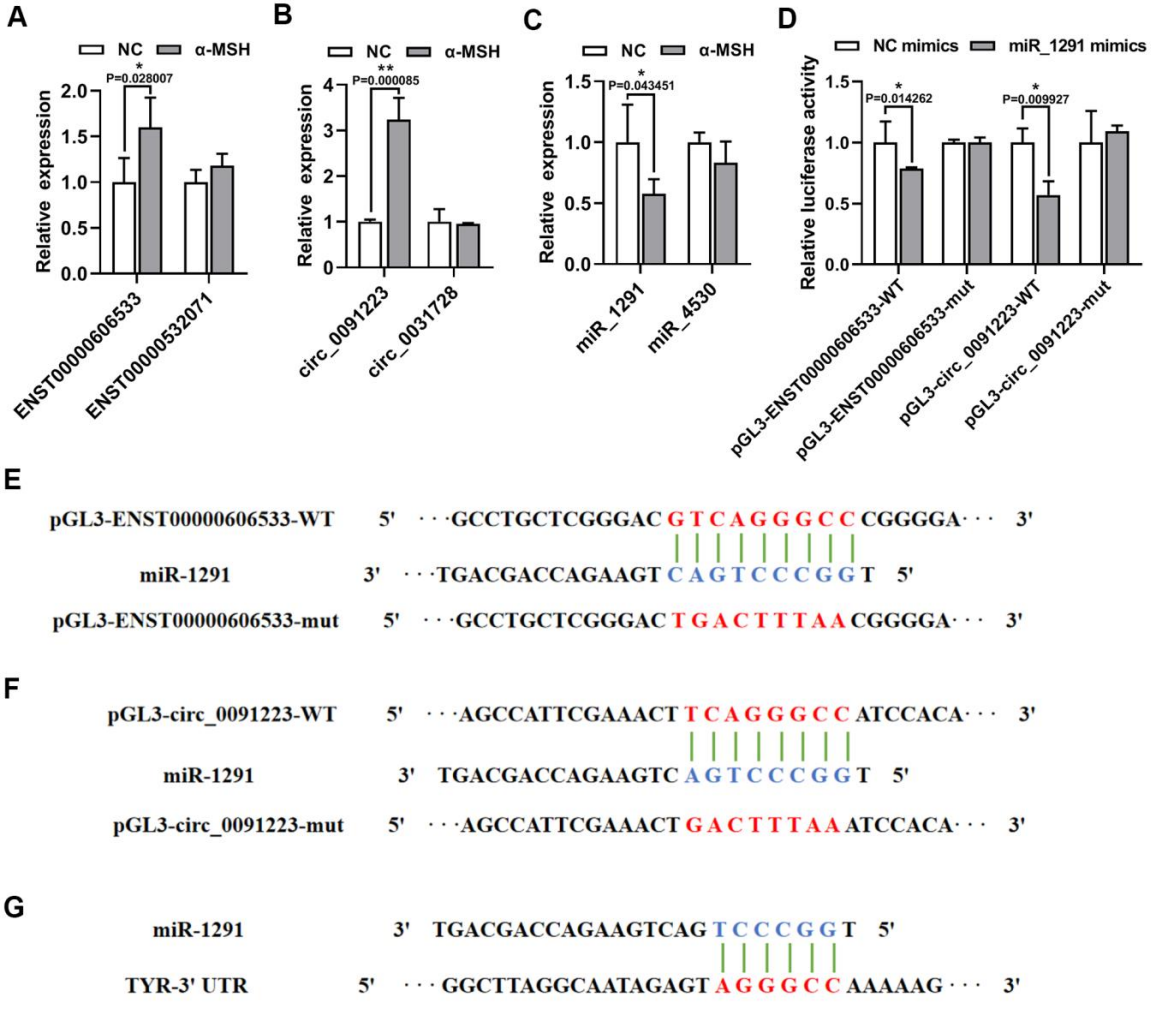
**α-MSH-treatment induced ENST00000606533 and circ_0091223 expression but inhibits miR-1291 expression.** (**A**–**C**) ENST00000606533, ENST00000532071, circ_0091223, circ_0031728, miR-1291, and miR-4530 levels were detected by qRT-PCR in MCs after 150 nM α-MSH treatment. (**D**) Luciferase reporter assay was used to detect the binding of miR-1291 to circ_0091223 and ENST00000606533. (**E**, **F**) Schematic model for wild type (WT) or mutant (mut) transcripts of ENST00000606533 or circ_0091223 luciferase reporters. (**G**) The binding site of miR-1291 with TYR predicted by TargetScan software.

As TYR is the key enzyme melanogenesis, we focused on the quaternary ceRNA network aimed at TYR. qRT-PCR results showed that ENST00000606533 and circ_0091223 significantly increased in α-MSH treated MCs compared with the NC MCs (Figure 4A, 4B), which was in accordance with the data from the microarray. While the expression of ENST00000532071 and circ_0031728 had no obvious change (Figure 4A, 4B). Besides, the expression of miR-1291 was significantly inhibited in α-MSH-treated MCs, while miR-4530 only had a slight decrease (Figure 4C). We further constructed luciferase reporters containing wild type and mutated putative binding sites of ENST00000606533 or circ_0091223 transcripts (Figure 4E, 4F), respectively. Luciferase reporter assays showed that the luciferase activities of ENST00000606533 or circ_0091223 wild type reporter were significantly reduced when transfected with miR-1291 mimics compared with control reporter or mutated luciferase reporter (Figure 4D). Moreover, the binding site of miR-1291 with TYR predicted by TargetScan software possessed the same site with which miR-1291 binding to ENST00000606533 or circ_0091223 (Figure 4G), so we did not further verify the binding relationship between miR-1291 and TYR by luciferase assay. These results confirmed that ENST00000606533 and circ_0091223 competitively bound to miR-1291, to affect the bind of miR-1291 with TYR as we predicted.

### Confirming the quaternary ceRNA network regulating TYR

**Figure 5:**
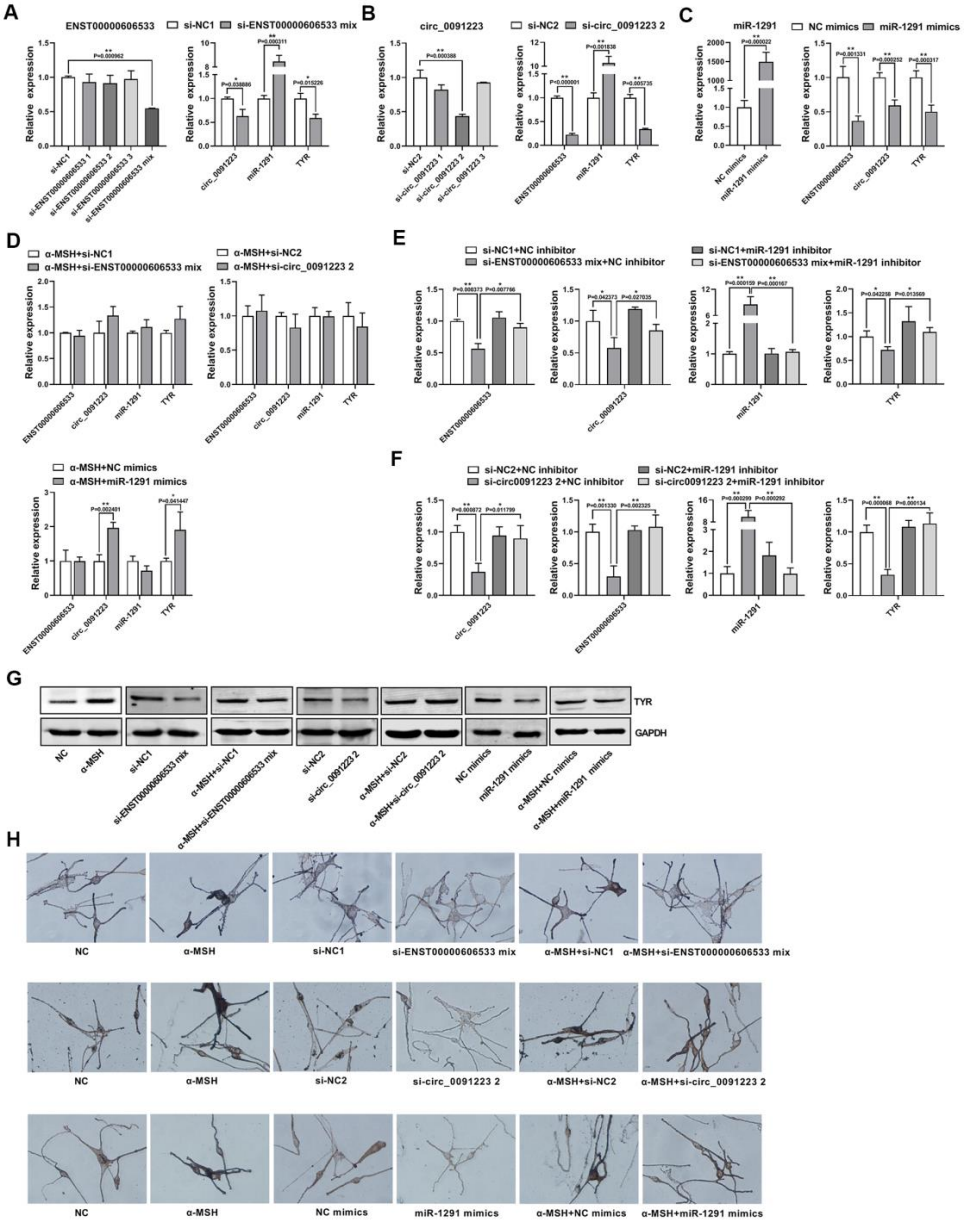
**Confirmation of the quaternary ceRNA network regulating TYR.** (**A**) After transfection with siRNAs of ENST00000606533 in MCs for 48 h, the transfection efficiency of si-ENST00000606533 mix (mixing the three siRNAs) was most significant, and the expression of circ_0091223, miR-1291, and TYR were detected by transfection with si-ENST00000606533 mix. (**B**) After transfection with siRNAs of circ_0091223 for 48 h, the transfection efficiency of si-circ_0091223 2 was most significant, and the expression of ENST00000606533, miR-1291, and TYR were detected by transfection with si-circ_0091223 2. (**C**) After transfection with miR-1291 mimics for 48 h, the transfection efficiency, the expression of ENST00000606533, circ_0091223, and TYR were detected. (**D**) After treated with α-MSH, the MCs were transfected with si-ENST00000606533 mix, or si-circ_0091223 2, or miR-1291 mimics, and the expression of ENST00000606533, circ_0091223, miR-1291, and TYR were detected. (**E**) After co-transfection with siRNAs of ENST00000606533 and miRNA_1291 inhibitor for 48 h, the expression of ENST00000606533, circ_0091223, miR-1291, and TYR were detected. (**F**) After co-transfection with siRNA of circ_0091223 and miR-1291 inhibitor for 48 h, the expression of ENST00000606533, circ_0091223, miR-1291, and TYR were detected. (**G**) After treated with α-MSH and/or si-ENST00000606533 mix, or si-circ_0091223 2, or miR-1291 mimics, the protein level of TYR were detected. (**H**) After treated with α-MSH and/or si-ENST00000606533 mix, or si-circ_0091223 2, or miR-1291 mimics, the Masson-Fontana melanin staining were detected.

To further confirm that ENST00000606533 and circ_0091223 can serve as ceRNAs to regulate TYR expression, we downregulated the expression of ENST00000606533 and circ_0091223 by siRNAs. The results showed that separate siRNA had poor interference effect on the expression of ENST00000606533, but mixed siRNAs could significantly inhibit the expression of ENST00000606533 (Figure 5A), so we chose mixed siRNAs in the subsequent experiments. si-ENST00000606533 mix could decrease the expression of circ_0091223 and TYR mRNA/protein level, while increase the expression of miR-1291 (Figure 5A, 5G). si-circ_0091223 2 could decrease the expression of ENST00000606533 and TYR mRNA/protein level, while increase the expression of miR-1291 (Figure 5B, 5G). We further upregulated the miR-1291 expression in MCs by transferring miR-1291 mimics. The results presented that miR-1291 mimics significantly decreased the expression of ENST00000606533, circ_0091223, and TYR mRNA/protein level (Figure 5C, 5G). Furthermore, we co-transfected the MCs with si-ENST00000606533 mix and miR-1291 inhibitor for 48 h and found that the miR-1291 inhibitor could reverse the inhibitory effect of si-ENST00000606533 mix on the expression of circ_0091223, ENST00000606533, miR-1291 and TYR (Figure 5E). Also, the inhibitory effect of si-circ_0091223 2 on the expression of circ_0091223, ENST00000606533, miR-1291 and TYR could be reversed by co-transfected with miR-1291 inhibitor (Figure 5F). The transfection efficiency of the miR-1291 inhibitor was showed in Supplementary Figure 3. And the si-ENST00000606533 mix, si-circ_0091223 2, and miR-1291 mimics could reverse the effect of α-MSH on ENST00000606533, circ_0091223, miR-1291, and TYR mRNA/protein level (Figure 5D, 5G). Moreover, the Masson-Fontana melanin staining showed that si-ENST00000606533 mix, si-circ_0091223 2, and miR-1291 mimics could inhibit the melanin production, and reverse the effect of α-MSH on melanin production (Figure 5H). These results indicated that ENST00000606533 and circ_0091223 could compete for binding to miR-1291 to upregulate TYR expression, forming the quaternary ceRNA network, then stimulating melanogenesis.

## DISCUSSION

Melanogenesis is regulated by multiple pathways in receptor dependent or independent mechanisms, which are driven by hormonal, autocrine, paracrine, or intracrine manner; one of the most important stimulus is α-MSH [21]. α-MSH controls diverse melanocytic functions, such as proliferation, eumelanin synthesis, and cytokine production [2]. In the skin, α-MSH is present in melanocytes and keratinocytes; thus, its cutaneous effects are presumably mediated via paracrine and/or autocrine mechanisms [22]. The paracrine and autocrine α-MSH performs its melanogenic action through several pathways, including cAMP/PKA/CREB/MITF, MAPK-ERK, and the Wnt signaling pathway [23–25]. Besides, some new mechanisms are involved in α-MSH-stimulated melanogenesis. One of them is autophagic tumor suppressor UVRAG, which is required for the dynamic integrity and pigmentation of melanosomes [26]. Moreover, microRNA-141-3p and microRNA-200a-3p can suppress α-MSH-induced melanogenesis via inhibiting MITF expression [27]. Hence, the mechanisms involved in α-MSH- induced melanogenesis is worthy of further study.

Recent studies have shown that ceRNAs are involved in various diseases, especially in cancers [8]. In some skin disease, such as psoriasis [28] and atopic dermatitis [29], lncRNAs or circRNAs also can act as ceRNAs and regulate these diseases via their interaction with disease-associated mRNAs. However, the functions of lncRNAs and circRNAs in α-MSH-induced melanogenesis are poorly understood. In our study, ceRNA microarray results showed that the expression profiles of lncRNAs and circRNAs are changed in α-MSH-treated melanocytes, suggesting that lncRNAs and circRNAs participate in α-MSH-induced melanogenesis. Our study also identified several lncRNA-miRNA-mRNA and circRNA-miRNA-mRNA ceRNA networks in α-MSH treated melanocytes, which can regulated differentially expressed melanogenesis-related genes (TYR, CTNNB1, IL6, CALM2, POMC, EDNRB, FZD7, WNT3, GNAO1, MAPK11, RAB38, FZD4, SOX6, and TYROBP). Since lncRNAs, circRNAs, and mRNAs can interact with miRNAs through MREs, we wondered if there is quaternary ceRNAs crosstalk involved in melanogenesis. The results of Venn diagram analysis and Cyto software (Vision 3.7.0) revealed that 13 quaternary ceRNA networks could regulate melanogenesis-related genes that differentially expressed in α-MSH treated melanocytes (Supplementary Figure 2 and Figure 3). Of course, these predicted quaternary ceRNA crosstalk requires further experimental confirmation.

**Figure 6:**
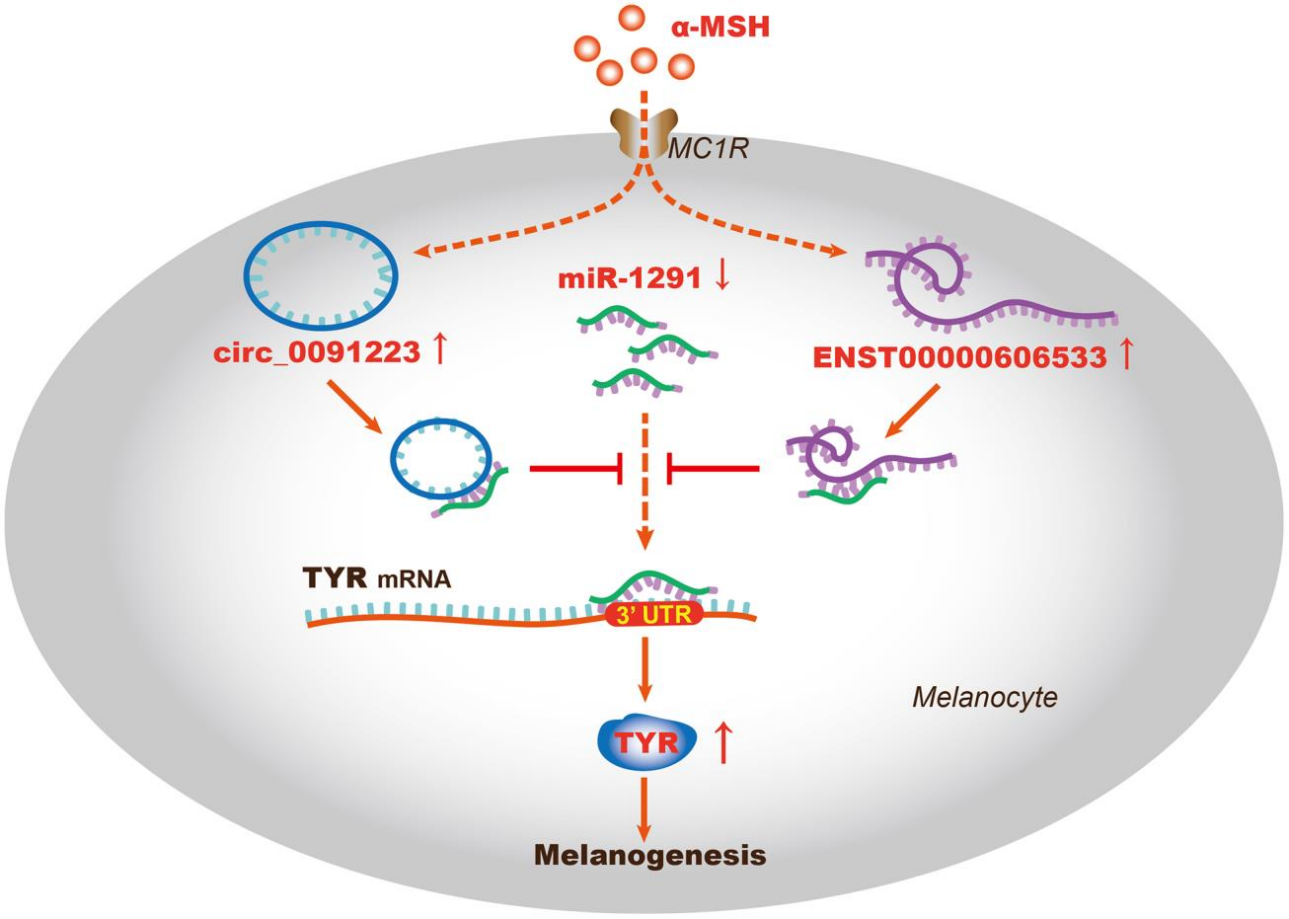
**The schematic diagram of the ceRNA mechanism in α-MSH-induced melanogenesis.** ENST00000606533 and circ_0091223 may function as ceRNAs to regulate TYR expression by sponging miR-1291, thus promoting α-MSH-induced melanogenesis in melanocytes.

TYR is the key enzyme that catalyzes L-tyrosine into L-DOPA, which is a rate-limiting step in melanin synthesis; thus, TYR can act as a marker that evaluates the function of melanogenesis [30]. As reported, TYR is regulated by transcription factors, histone modification related proteins, ubiquitination related proteins, miRNAs, and lncRNAs. For example, MITF is the most important transcription factor to promote TYR expression [31], the histone acetyl transferase (HAT) p300/CBP can mediate H3K27 acetylation, which further regulates TYR expression through the interaction with MITF [30]. Besides, TYR protein can be ubiquitinated and degraded by proteasome complex (E1, E2, and E3) of ER [32, 33]. Moreover, miR-125b can decrease TYR expression in human melanocytes and pigmented tissue [34], LncRNA H19 and UCA1 can directly or indirectly regulate TYR expression [14, 15]. As we know, the ceRNA hypothesis proposes that lncRNAs, circRNAs, and mRNAs competing for binding the miRNAs and regulating the expression of each other, which forms a complex post-transcriptional regulatory network. Since that miRNA and lncRNA are related to TYR, does the ceRNA regulatory mechanism play a role in regulating α-MSH-induced TYR expression? Our study had identified the possible quaternary ceRNA network regulating TYR (Figure 3A). So we further confirmed the hypothesistaking TYR as an example. We firstly found that the expression of ENST00000606533, circ_0091223, and TYR was upregulated, miR-1291 was downregulated in melanocytes after α-MSH treatment. Simultaneously, we designed ENST00000606533 or circ_0091223 luciferase reporter plasmids for miR-1291, and found that miR-1291 mimics reduced the luciferase activity of ENST00000606533 and circ_0091223 luciferase reporter, respectively. Besides, the silence of ENST00000606533 and circ_0091223 could increase miR-1291 expression and decrease TYR expression. miR-1291 mimics could decrease the expression of ENST00000606533, circ_0091223, and TYR. Further rescue experiments showed that miR-1291 inhibitor significantly attenuated the effects of ENST00000606533 and circ_0091223 silencing on TYR, suggesting that ENST00000606533 and circ_0091223 may function as ceRNAs to regulate α-MSH-induced TYR by sponging miR-1291 (Figure 6). The identification of “ENST00000606533/circ_0091223- miR-1291-TYR” axis expands the understanding of the underlying mechanism of α-MSH-induced melanogenesis in melanocytes, which also provides novel research directions for skin pigmentation disease. These results also support that ENST00000606533, circ_0091223, and miR-1291 may play an important role in melanogenesis.

The mechanism of α-MSH promoting ENST00000606533 and circ_0091223 expression in melanocytes is unclear. At present, the expression of lncRNAs and circRNAs are mainly regulated at three different levels: epigenetic regulation (DNA methylation, histone modification, chromatin remodeling and RNA modification), transcriptional regulation (the interaction with transcription factors and single nucleotide polymorphism), and post-transcriptional regulation (the interaction with miRNAs, RNA-binding proteins, and general splicing factors) [35, 36]. α-MSH can promote melanogenesis via binding to its receptor and driving downstream signaling pathways, especially cAMP/PKA signaling pathway. Multiple studies have shown that cAMP/PKA pathway can not only induce the expression of some transcription factors, such as Signal Transducer And Activator Of Transcription 5B (STAT5B), Nuclear Receptor Subfamily 4 Group A Member 3 (NR4A3), c-Fos, and CREB [37, 38], but also drive the epigenetic regulation and post-transcriptional regulation. For example, PKA can phosphorylate histone demethylases PHF2, which can bind and demethylate DNA-binding protein ARID5B, thus affecting the expression of downstream genes [39]. Besides, cAMP/PKA pathway can promote apoptosis through the post-transcriptional regulation such as lysosomal, ribosomal, Golgi-related functions and RNA splicing [40–42]. Hence, α-MSH may increase the expression of ENST00000606533 and circ_0091223 through one of above mechanism, which needs further investigation in the follow-up study. There are some limitations in this study. For example, our study is based on *in vitro* experiments and lacking *in vivo* evidence, such as clinical specimens and animal experiments. We have confirmed that the ceRNA regulatory network can regulate TYR in α-MSH treated melanocytes, but other melanogenesis-related genes included in the networks are not explored, which might be investigated in the future.

## CONCLUSION

Our study reveals that ceRNA regulatory network is a new mechanism involved in α-MSH-induced melanogenesis. The role of ENST00000606533/circ_0091223- miR-1291-TYR quaternary ceRNA network in melanogenesis is first identified. These findings provide a novel insight into understanding the mechanism of melanogenesis and suggest potential therapeutic targets for skin pigmentation disorder.

## MATERIALS AND METHODS

### Cell lines isolation and culture

The MCs were extracted from the prepuce, which was donated by the adolescent males who were circumcised in the Department of Urology Surgery, Third Xiangya Hospital, Central South University. The consent of patients was obtained and approved by the Ethics Committees of Third Xiangya Hospital, Central South University. The samples (four donators) were firstly immersed in Dulbecco’ s Phosphate-Buffered Saline (DPBS; Thermo Fisher Scientific, Inc., Waltham, MA, USA) containing Penicillin/Streptomycin/Amphotericin B sterile solution (Solarbio, Beijing, China) at 4° C for 4 h and washed three times with DPBS containing Penicillin/Streptomycin/Amphotericin B sterile solution. Following removal of subcutaneous tissue, the samples were cut into pieces and digested with Dispase II (D4693-1G, Sigma-Aldrich, Shanghai, China) at 4° C for 20 h to separate intact epidermis from dermis. After that, the epidermis was cut into pieces and digested with trypsin. MCs were collected, washed, and then incubated with 254 medium (#M-254-500, Gibco, USA) containing 5% fetal bovine serum (FBS, Biological Industries, Israel) and 1% HMGS (# S0025, Gibco, USA) in cell culture flasks at 37° C with 5% CO_2_. MCs in logarithmic phase from the third to fifth passage were used for experiments, and were cultured in cell culture dishes or cell culture plates according to the need of every experiment. The cells from donators used individually.

### Oligonucleotides and transfection

siRNAs of ENST00000606533 (si-ENST00000606533 1, si-ENST00000606533 2, and si-ENST00000606533 3) and the corresponding negative control (si-NC1) were synthesized by Ribobio (Guangzhou, China); siRNAs of circ_0091223 (si-circ_0091223 1, si-circ_0091223 2, and si-circ_0091223 3) and the corresponding negative control (si-NC2) were synthesized by GenePharma (Shanghai, China); miR-1291 mimics and inhibitor were synthesized by GenePharma, and the corresponding negative control were simply known as NC mimics and NC inhibitor, respectively. The stored concentration of all the siRNAs, mimics and inhibitors was 20 μM. When the MCs were seeded in 6-well plates or 60 mm cell culture dishes for transfection, and allowed to grow to 60%-80%, the added volume of siRNAs, mimics and inhibitors were 10 μL or 30 μL. And cells were transfected using Lipofectamine 2000 (Invitrogen, CA, USA) and harvested for the experiment after 48 h. Oligonucleotide sequence is listed in Supplementary Table 1, but Ribobio refused to provide the oligonucleotide sequence of si-NC1.

### Microarray analysis

The MCs were seeded in the 60 mm cell culture dish and allowed to grow to 60%-80%, and were stimulated by α-MSH for 24 h. Then total RNAs were extracted from the MCs with and without the treatment of 150 nM α-MSH (M4135, Sigma-Aldrich, USA) by TRIzol reagent (Ambion, USA). RNA samples were then used to generate fluorescence-labeled complementary RNA (cRNA) targets for the SBC-ceRNA array (Human 4×180K, designed by Shanghai Biotechnology Corporation, and made by Agilent Technologies), which contains 88,371 circRNA probes, 68,423 lncRNA probes, and 18,853 mRNA probes. Raw data were normalized by the Quantile algorithm, limma package in the R program. Significant differentially expressed transcripts were screened by fold change (linear) ≥ 2.

### Bioinformatics analysis

We conducted gene ontology (GO) analysis and Kyoto Encyclopedia of Genes and Genomes (KEGG) enrichment analysis to investigate the roles of all significant differentially expressed mRNAs, as previously screened. Briefly, GO analysis was applied to elucidate genetic regulatory networks of interest by forming hierarchical categories according to the biological processes, cellular component, and molecular functions aspects of the differentially expressed genes (DEGs) (http://geneontology.org/). The selection criterion is the count of genes that differ in a term/GO: ≥ 10, the p_value < 0.05, and the enrich factor value ≥ 1.65. According to the value of enrich factors in descending order of size, melanosome/GO (TYR, ATP6V1B2, ERP29, CCT4, RAN, HSPA8, RAB38, ANXA2, RPN1, SEC22B, TYROBP, and ATP1B3) comes in at number nine. KEGG pathway analysis were performed to explore significant pathways associated with the DEGs (http://www.genome.jp/kegg/). The selection criterion is the count of genes that differ in a term/pathway: > 5. According to the value of enrich factors in descending order of size, melanogenesis/pathway (EDNRB, GNAO1, FZD7, POMC, TYR, CTNNB1, FZD4, CALM2, and WNT3) comes in at number nine. Then combined the two analysis and other reports [18–20], we selected DEGs, which is related to melanogenesis (TYR, Catenin Beta 1 (CTNNB1), Interleukin 6 (IL6), Calmodulin 2 (CALM2), Proopiomelanocortin (POMC), Receptor Type B (EDNRB), Frizzled Class Receptor 7 (FZD7), Wnt Family Member 3 (WNT3), Protein Subunit Alpha O1 (GNAO1), Mitogen-Activated Protein Kinase 11 (MAPK11), Member RAS Oncogene Family (RAB38), Endothelin G Frizzled Class Receptor 4 (FZD4), SRY-Box Transcription Factor 6 (SOX6), and TYRO Protein Tyrosine Kinase Binding Protein (TYROBP)). Differentially expressed lncRNAs were identified by the screened criteria (normalized signal value > 2 and fold change > 5). Differentially expressed circRNAs were identified by the screening criteria (normalized signal value > 1.5 and fold change > 5). miRNA binding sites were predicted using TargetScan 7.1 and miRanda v3.3a. miRNAs associated with the screened lncRNAs were filtered through relatively high scores (≧160). And each screened circRNAs corresponded to the top five miRNAs with high-binding sites.

### Quantitative Real-Time PCR (qRT-PCR)

The MCs were seeded in the 6-well plates and allowed to grow to 60%-80%, and were stimulated by α-MSH and/or siRNA for 24 h or 48 h. Then the total RNA of MCs was extracted by TRIzol Reagent (Invitrogen, Thermo Fisher Scientific, USA). The miRNA was extracted by the E.Z.N.A.® Micro RNA Kit (R6842-01, Omega Bio-Tek, USA). SYBR qPCR Mix (#QPS-201T, TOYOBO) was used for qRT-PCR analysis. All reactions were run on a real-time PCR instrument (Roche LightCycler480II, Germany). The conditions for qRT-PCR were consisted of 40 cycles of 95° C for 15 sec, 57° C for 15 sec, and 68° C for 20 sec, after an initial denaturation step (95° C for 60 sec). The primers used in this study are summarized in Supplementary Table 2. All lncRNA, circRNA and mRNA expression data were normalized to GAPDH, and miRNAs were normalized to U6.

### Western blot analysis

The MCs were seeded in the 60 mm cell culture dish and allowed to grow to 60%-80%, and were stimulated by α-MSH and/or siRNA for 48 h. Then, the total protein of cells was extracted using RIPA lysis buffer with a cocktail of proteinase inhibitors (Roche, Mannheim, Germany) and a cocktail of phosphatase inhibitors (Roche, Mannheim, Germany) according to its protocol. 30 μg total protein was separated by 10% SDS-PAGE and transferred onto a PVDF membrane. After blocking by 0.1% BSA, the membranes were incubated with primary antibody TYR (#ab180753, Abcam, Cambridge, UK) and Glyceraldehyde-3-Phosphate Dehydrogenase (GAPDH, AP0066, Bioworld Technology, Inc., Minnesota, USA) overnight at 4° C and followed by an incubation period of 1 h at room temperature with secondary antibody (1:10000, LI-COR Biosciences, USA). Bands were detected by the enhanced LI-COR Odyssey infrared imaging system (LI-COR Biosciences, NE, USA).

### Luciferase reporter assay

Bioinformatics tools (microRNA.org) were used to predict the miR-1291 binding sites of circ_0091223 and ENST00000606533. 5×10^4 MCs were seeded in 24-well plates and cotransfected with a mixture of 180 ng luciferase reporter vectors (empty pmirGLO-NC, pmirGLO-circ_0091223-WT or pmirGLO-circ_0091223-mut; empty pmirGLO-NC, pmirGLO-ENST00000606533-WT or pmirGLO-ENST00000606533-mut), 18 ng Renila luciferase reporter vectors (pRL-TK) (Genechem, Shanghai, China), and miR-1291 mimics (GenePharma) at the indicated concentration. After 48 h, the luciferase activity was measured with a multifunction microplate reader (PerkinElmer EnVision Xcite, UK). The luciferase values were normalized to the corresponding Renila luciferase values, and then the fold changes were calculated. The vectors construction sequence are showed in Figure 4E, 4F.

### Masson-Fontana melanin staining

After α-MSH treatment and/or siRNAs transfection for 48 h, MCs were washed with PBS and fixed with 4% paraformaldehyde for 30-60 min. Then, the cells were treated with Fontana ammonia silver solution, placed in an oven at 56° C for 30-40 min, and washed 5 times with distilled water for 1 min each time. Next, the cells were placed in the hypo solution at room temperature for 5 min and washed by tap water for 5 min. The images were captured using an inverted fluorescence microscope (Olympus, IX73, Japan).

### Statistical analysis

Statistical analyses were performed using the software GraphPad Prism 8.0.2, and consisted of analysis of variance followed by Student’ s t-test when comparing two experimental groups. *P* < 0.05 was considered statistically significant, and * was indicated *P* < 0.05, ** was indicated *P* < 0.01.

## Supplementary Material

Supplementary Figures

Supplementary Tables
