# Supplementary material for: Identification of the ceRNA networks in α-MSH-induced melanogenesis of melanocytes
Source: Aging (Albany NY). 2020 Dec 14;13(2):2700–26. doi: 10.18632/aging.202320 (PMC7880406; doi:10.18632/aging.202320)
Supplement: Supplementary Figures [file aging-13-202320-s001.pdf]

SUPPLEMENTARY FIGURES

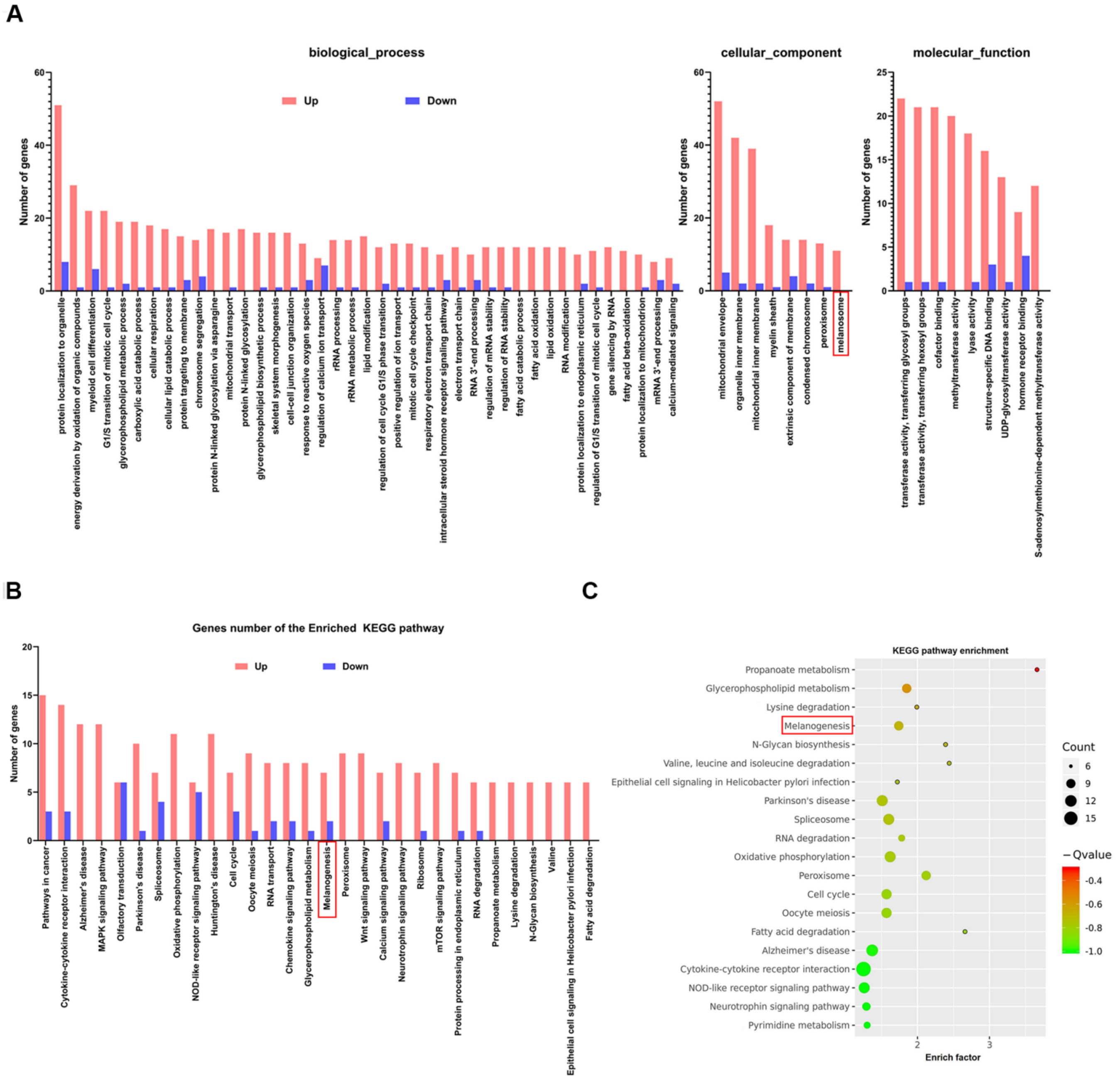

Supplementary Figure 1. GO and KEGG pathway enrichment analysis. (**A**) GO analysis of differentially expressed genes, which were screened by the number of genes ≥10, and enrich factors and ≥1.65; (**B**, **C**) The KEGG pathway enrichment of differentially expressed genes, which were screened by the number of genes ≥5, and the top 30 genes by qvalue.

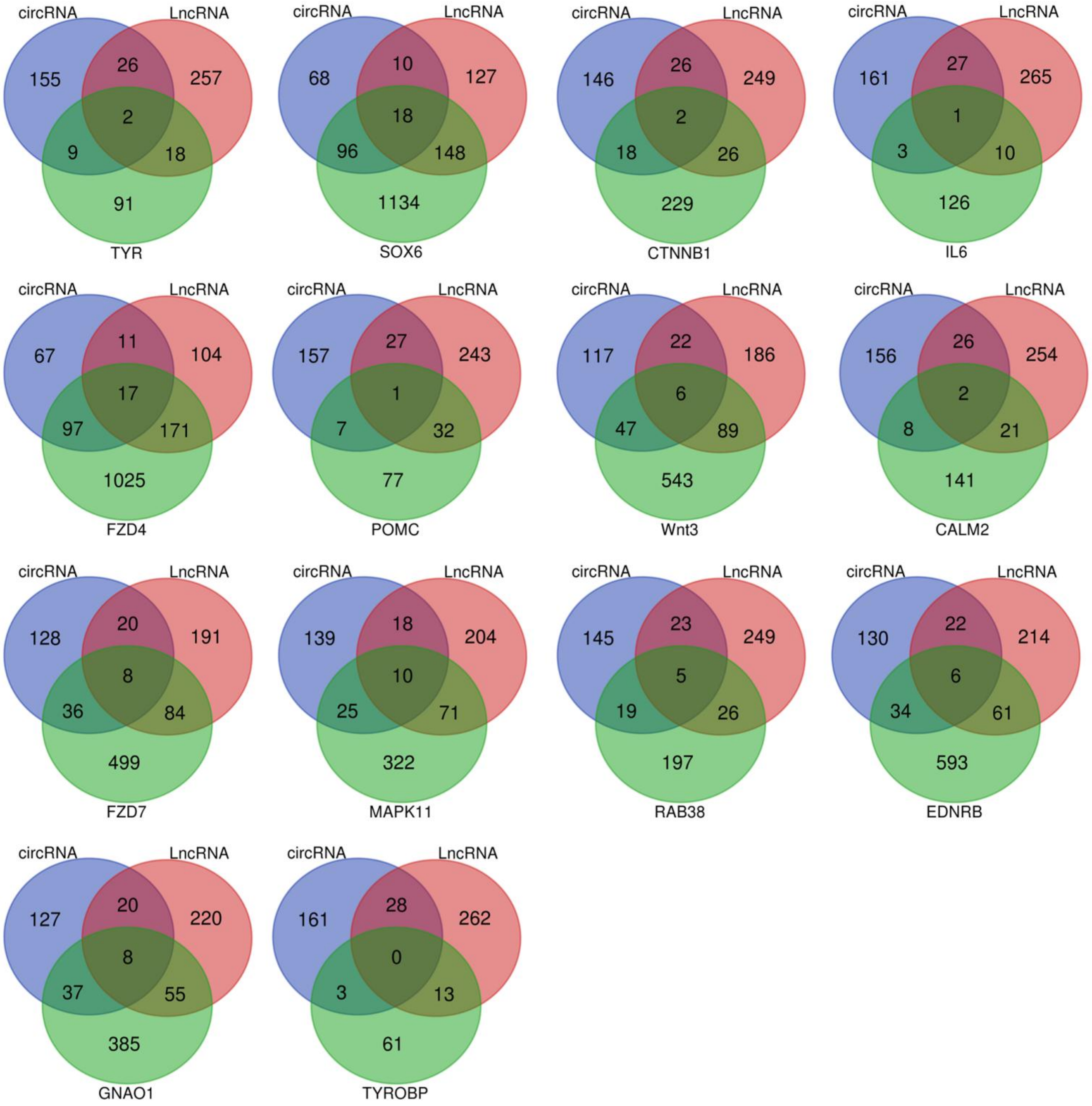

Supplementary Figure 2. Venn diagram analysis was used to screen the potential quaternary ceRNA network focused on melanogenesis related mRNAs (TYR, SOX6, CTNNB1, IL6, FZD4, POMC, WNT3, CALM2, FZD7, MAPK11, RAB38, EDNRB, and GNAO1).

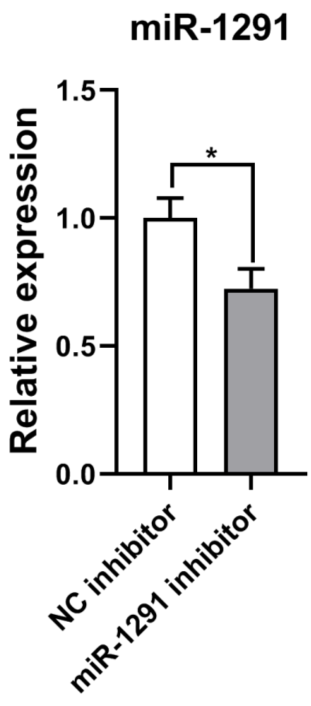

Supplementary Figure 3. After transfection with miR-1291 inhibitor for 48 h, the transfection efficiency were detected by qRT-PCR.
