# Supplementary material for: Identification of the ceRNA networks in α-MSH-induced melanogenesis of melanocytes
Source: Aging (Albany NY). 2020 Dec 14;13(2):2700–26. doi: 10.18632/aging.202320 (PMC7880406; doi:10.18632/aging.202320)
Supplement: Supplementary Tables [file aging-13-202320-s002.pdf]

SUPPLEMENTARY TABLES

Supplementary Table 1. Oligonucleotide sequence.

siRNA	Target sequence (5'-3')
si-ENST00000606533 1	CACAGCAAGATGCAGACAA
si-ENST00000606533 2	CCACACCTATACCTTCTCA
si-ENST00000606533 3	GCCACCAGGTATCTTTACA
si-NC2	UUCUCCGAACGUGUCACGUTT
si-circ_0091223 1	GGAAUGUCAUUGAGUGUGATT
si-circ_0091223 2	AUGUCAUUGAGUGUGAUUGTT
si-circ_0091223 3	CAUUGAGUGUGAUUGGCCUTT
NC inhibitors	CAGUACUUUUGUGUAGUACAA
miR-1291 inhibitors	ACUGCUGGUCUUCAGUCAGGGCCA
NC mimics	UUGUACUACACAAAAGUACUG
miR-1291 mimics	UGGCCCUGACUGAAGACCAGCAGU

Supplementary Table 2. The primers sequence in the study.

Genes	Forward sequence (5'-3')	Reverse primer (5'-3')
ENST00000532071	GGAGACCTGATGTCCCAATT	GTAAGGCTGTGAACCGTGTA
ENST00000606533	GCATCATCGCTTTCAACTTCG	GAATAGAGCCCACGGTCCTG
circ_0031728	GACTGCTGGAGATTCTTACAAC	CCCCAGAGGAACGCAAAGGT
circ_0091223	ATGTCATTGAGTGTGATTGGCC	GAATGGCTTGAATCACCTTG
miR_4530	GTCGTATCCAGTGCAGGGTCCGAGGTATTCGCACTGGATACGACCGCTCC	CGCGCCCAGCAGGACG
miR-1291	GTCGTATCCAGTGCAGGGTCCGAGGTATTCGCACTGGATACGACACTGCT	CGTGGCCCTGACTGAAGACC
TYR	TCAGCCCAGCATCATTCTTC	GGCATCCGCTATCCCAGTAA
MITF	AAATACGTTGCCTGTCTCGG	TGTTGGGAAGGTTGGCTGGA
TYRP1	ACCAGAGGGTTCTCATAGTCAG	TTCTCAAATTGTGGCGTGTT
DCT	GGGCAGCGAGACCAGACGAT	TTGGCAATTTCATGCTGTTTCTTC
RAB27A	GTAGTGAAAGAGGAGGAAGC	TCATTATCAGGTCCAGAAGC
MYO5A	GCCCGATGCTATGCTAAGTT	GCCAAGAAGCCTCGCAAGTA
U6	GCTCGCTTCGGCAGCACATAT	ATATGGAACGCTTCACGAATTTGC
GAPDH	CTCTGCTCCTCCTGTTCGAC	GCCCAATACGACCAAATCC

Supplementary Table 3. The predicted miRNA of screened lncRNA in α-MSH-induced melanogenesis.

LncRNA	Predicted miRNA
ENST00000532071	miR-6851-5p, miR-484, miR-1291, miR-6817-5p, miR-3120-5p, miR-5189-5p, miR-663b, miR-6847-3p, miR-6511b-5p, miR-5695, miR-6799-3p, miR-6772-3p, miR-4459, miR-4691-5p, miR-4421, miR-7158-5p, miR-6865-5p
NR_040100	miR-7851-3p, miR-1268b, miR-675-5p, miR-1268a, miR-4516, miR-3157-5p, miR-30c-2-3p, miR-6765-3p, miR-4466, miR-5096, miR-619-5p, miR-6760-5p, miR-4459, miR-6755-5p, miR-29b-2-5p, miR-2467-3p, miR-1304-5p, miR-373-3p, miR-106b-5p, miR-4713-5p, miR-1910-3p, miR-4439, miR-1233-5p, miR-3192-5p, miR-4518, miR-888-3p, miR-20b-5p, miR-93-5p, miR-6884-5p, miR-6780a-5p, miR-4695-5p, miR-17-5p, miR-20a-5p, miR-4259, miR-2681-5p, miR-6513-3p, miR-6813-5p, miR-6831-5p, miR-4493, miR-6089, miR-665, miR-3154, miR-4700-5p, miR-4469, miR-4725-3p, miR-4667-5p
ENST00000316807	miR-6849-3p, miR-4722-5p, miR-4652-5p, miR-4433a-3p, miR-3657, miR-324-3p, miR-7160-5p, miR-1913, miR-1273g-3p, miR-1254, miR-7851-3p, miR-4738-3p, miR-4699-5p, miR-1285-3p, miR-4733-3p, miR-6870-3p, miR-4649-5p, miR-4776-3p, miR-4708-3p, miR-573, miR-4439, miR-665, miR-3129-5p, miR-6757-3p, miR-4280, miR-4646-5p, miR-3135b, miR-4494, miR-922, miR-7703, miR-542-3p, miR-4421, miR-3174, miR-6884-5p, miR-181b-5p, miR-431-5p, miR-4515, miR-619-5p
ENST00000606533	miR-6791-3p, miR-7706, miR-4530, miR-508-5p, miR-1281, miR-6829-3p, miR-2467-5p, miR-619-5p, miR-143-5p, miR-3620-5p, miR-6801-3p, miR-3619-5p, miR-1291, miR-4673, miR-1587, miR-6810-3p, miR-6825-5p, miR-6791-5p, miR-5095, miR-1304-3p, miR-214-3p, miR-378a-5p, miR-4430, miR-939-3p, miR-7113-3p, miR-17-5p, miR-6811-5p, miR-3135b, miR-4538, miR-212-5p, miR-504-3p, miR-7160-5p, miR-20b-5p, miR-6747-3p, miR-3155a, miR-6763-5p, miR-5189-5p, miR-3943, miR-6787-5p, miR-658, miR-7114-5p, miR-8089, miR-4763-3p, miR-760, miR-4260, miR-4692, miR-6799-3p, miR-330-5p, miR-6883-5p, miR-3678-3p
lnc-CYP4F22-1:1	miR-6808-5p, miR-1285-3p, miR-1304-5p, miR-1827, miR-3187-5p, miR-6720-5p, miR-6884-5p, miR-6771-5p, miR-612, miR-6780a-5p, miR-5189-5p, miR-6086, miR-6779-5p, miR-661, miR-766-3p, miR-1273h-5p, miR-3064-5p, miR-4646-5p, miR-4491, miR-6089, miR-367-3p, miR-6820-3p, miR-1273e, miR-3622a-5p, miR-4802-3p, miR-6744-5p, miR-7703, miR-1268b, miR-1268a, miR-7157-3p
ENST00000587528	miR-654-5p, miR-6749-5p, miR-1908-3p, miR-92b-5p, miR-541-3p, miR-6825-5p, miR-4674, miR-9500, miR-3185, miR-6894-5p, miR-3960, miR-181a-2-3p, miR-8089
NR_031650	miR-578
lnc-LNPEP-2:1	miR-6878-5p, miR-6871-5p, miR-6779-3p, miR-4518, miR-3127-5p, miR-1266-5p, miR-1233-3p
ENST00000530955	miR-6833-3p, miR-6511b-5p, miR-4774-5p, miR-3670, miR-4524a-5p, miR-150-3p, miR-5001-5p, miR-4704-3p, miR-6832-3p, miR-4481, miR-6510-5p, miR-3620-5p, miR-4692, miR-6511a-5p, miR-4524b-5p, miR-8055, miR-4743-3p, miR-6734-3p, miR-892b, miR-6873-3p
ENST00000533322	miR-3187-5p, miR-612, miR-6856-3p, miR-6860, miR-6721-5p, miR-4758-3p, miR-6890-5p, miR-6812-5p, miR-6819-5p, miR-541-3p, miR-614, miR-4254, miR-378c, miR-1298-3p, miR-1251-5p, miR-212-5p, miR-3929
NR_104161	miR-378g, miR-7851-3p, miR-661, miR-939-3p, miR-6807-5p, miR-612, miR-3128, miR-3187-5p, miR-4797-3p, miR-4716-5p, miR-550b-2-5p, miR-4743-3p, miR-4525, miR-3652, miR-532-5p, miR-3137, miR-508-5p, miR-7843-5p, miR-4505
ENST00000414633	miR-4518, miR-875-3p, miR-490-3p, miR-345-5p, miR-619-3p, miR-5008-3p, miR-6822-3p, miR-1247-5p, miR-6737-3p, miR-3617-3p
ENST00000599817	miR-4656, miR-378g, miR-3620-5p, miR-1587, miR-6729-5p, miR-4505, miR-6758-5p, miR-6783-3p, miR-1343-3p, miR-939-3p
lnc-ELAVL2-1:2	miR-4686, miR-4436a, miR-4715-5p, miR-4668-3p, miR-1305, miR-185-5p, miR-106b-5p, miR-6881-3p, miR-4420, miR-6766-5p, miR-5739, miR-4293, miR-3173-3p, miR-29a-3p, miR-6720-5p, miR-4312, miR-2355-5p, miR-543
lnc-RBMY1J-6:1	miR-3591-3p, miR-141-5p, miR-7156-3p, miR-3612, miR-4308, miR-412-3p, miR-4742-3p, miR-6754-3p, miR-5193, miR-3679-5p, miR-4794

lnc-KIF20A-1:1	miR-550a-5p, miR-2110, miR-106a-5p, miR-17-5p, miR-20a-5p, miR-1254, miR-550a-3-5p, miR-3116, miR-33b-3p
lnc-TRIM37-1:2	miR-6511a-3p, miR-6824-3p, miR-4758-3p, miR-6837-3p, miR-6764-3p, miR-21-5p, miR-6849-3p, miR-765, miR-1224-3p, miR-584-3p
NR_104003	miR-6758-5p, miR-4644, miR-4510, miR-4747-5p, miR-4533, miR-6780b-5p, miR-3619-5p, miR-646, miR-5196-5p, miR-766-3p, miR-761, miR-214-3p, miR-6760-5p, miR-5186, miR-486-5p, miR-181a-2-3p, miR-3145-5p, miR-8081, miR-150-5p, miR-184, miR-6862-3p, miR-6089, miR-4767, miR-6510-5p, miR-4667-3p, miR-6739-5p, miR-3162-3p, miR-935, miR-4679, miR-6127, miR-6845-3p, miR-6720-5p, miR-4722-5p, miR-34a-5p, miR-892c-3p
lnc-BTBD19-1:1	miR-4789-5p, miR-5588-3p, miR-7106-3p, miR-425-5p, miR-4659a-5p, miR-4730, miR-7113-3p, miR-4433a-5p, miR-619-5p, miR-486-5p

Supplementary Table 4. The predicted miRNA of screened circRNA in α-MSH-induced melanogenesis.

CircRNA	Predicted miRNA
circ_0031794	miR-519d-5p, miR-4694-5p, miR-6744-5p, miR-8082, miR-4534
circ_0035909	miR-4436b-5p, miR-942-5p, miR-6509-3p, miR-4659a-3p, miR-4659b-3p
circ_0074400	miR-3167, miR-876-5p, miR-1972, miR-4691-3p, miR-4659a-5p
circ_0066944	miR-338-3p, miR-3972, miR-1202, miR-5088-3p, miR-3194-5p
circ_0030056	miR-615-5p, miR-4290, miR-3189-3p, miR-491-5p, miR-3138
circ_0054509	miR-641, miR-3617-5p, miR-4756-3p, miR-6728-5p, miR-6072
circ_0015211	miR-4677-5p, miR-578, miR-629-3p, miR-22-5p, miR-2116-5p
circ_0054701	miR-1226-3p, miR-6828-3p, miR-3940-3p, miR-5196-3p,
circ_0068095	miR-6509-3p, miR-9-3p, miR-181a-2-3p, miR-4422, miR-4504
circ_0037543	miR-7106-5p, miR-4739, miR-1827, miR-1273g-3p, miR-149-3p
circ_0084506	miR-519e-5p, miR-515-5p, miR-519d-5p, miR-5695, miR-433-3p
circ_0002626	miR-5089-3p, miR-148a-3p, miR-148b-3p, miR-152-3p, miR-8080
circ_0083993	miR-3191-5p, miR-455-3p.1, miR-139-3p, miR-3944-5p, miR-4420
circ_0025748	miR-4659a-3p, miR-4659b-3p, miR-4677-5p, miR-2116-5p, miR-22-5p
circ_0069815	miR-4659a-3p, miR-4659b-3p, miR-4778-3p, miR-130b-5p, miR-3942-3p
circ_0053692	miR-4793-3p, miR-1288-3p, miR-24-3p, miR-6842-3p, miR-1269a
circ_0067366	miR-451b, miR-6817-5p, miR-640, miR-4769-3p, miR-4727-5p
circ_0059675	miR-4454, miR-876-3p, miR-562, miR-31-5p, miR-548v
circ_0091223	miR-197-3p, miR-455-3p.2, miR-8056, miR-1291, miR-6775-3p
circ_0031463	miR-4778-3p, miR-5193, miR-660-3p, miR-219a-5p, miR-4782-3p
circ_0053508	miR-4691-5p, miR-6792-3p, miR-3653-5p, miR-6842-3p, miR-4267
circ_0020240	miR-4672, miR-3913-5p, miR-3122, miR-3120-3p, miR-6768-5p
circ_0058701	miR-3915, miR-5582-5p, miR-1910-5p, miR-1224-5p, miR-4689
circ_0026068	miR-328-5p, miR-6885-5p, miR-4326, miR-518c-5p, miR-6780a-3p
circ_0015226	miR-6884-5p5, miR-485-5p5, miR-39753, miR-31883, miR-2467-5p3
circ_0007726	miR-4645-3p, miR-612, miR-5189-5p, miR-1285-3p, miR-6860
circ_0050401	miR-6836-3p, miR-4772-5p, miR-662, miR-383-5p.2, miR-4281

circ_0058630	miR-509-3-5p, miR-509-5p, miR-4418, miR-197-3p, miR-760
circ_0083902	miR-670-5p, miR-3677-5p, miR-4786-3p, miR-622, miR-642b-5p
circ_0088002	miR-5006-5p, miR-766-3p, miR-1202, miR-3972, miR-4533
circ_0079872	miR-432-3p, miR-8058, miR-5009-5p, miR-3074-5p, miR-7152-5p
circ_0068584	miR-4738-5p, miR-4701-5p, miR-588, miR-4435, miR-4709-5p
circ_0001808	miR-3977, miR-95-5p, miR-1184, miR-4288, miR-632
circ_0021740	miR-6748-3p, miR-3614-3p, miR-4778-3p, miR-4421, miR-5699-3p
circ_0073019	miR-6873-3p, miR-6833-3p, miR-4768-5p, miR-545-3p, miR-6817-3p
circ_0031728	miR-4677-3p, miR-4530, miR-4713-5p, miR-766-3p, miR-3135b
circ_0053546	miR-5193, miR-660-3p, miR-4326, miR-4267, miR-3922-5p
circ_0014138	miR-5193, miR-660-3p, miR-1248, miR-6868-3p, miR-1237-3p
circ_0055832	miR-3663-5p, miR-455-3p.2, miR-4778-3p, miR-6744-3p, miR-4757-5p
circ_0047279	miR-4800-3p, miR-6877-3p, miR-6890-3p, miR-3713, miR-4780
circ_0077787	miR-578, miR-1539, miR-4691-5p, miR-6792-3p, miR-140-3p.1
circ_0072730	miR-29a-5p, miR-452-3p, miR-653-3p, miR-5703, miR-4516
circ_0073464	miR-4493, miR-6126, miR-605-3p, miR-4753-5p, miR-1825
circ_0030604	miR-6890-3p, miR-7977, miR-186-3p, miR-483-3p.1, miR-4436b-3p

Supplementary Table 5. The predicted miRNA of screened mRNA in α-MSH-induced melanogenesis.

mRNA	Predicted miRNA
CALM2	miR-93-3p, miR-874-5p, miR-8086, miR-7109-3p, miR-7108-5p, miR-6867-3p, miR-6828-3p, miR-6810-3p, miR-6802-3p, miR-6801-3p, miR-6778-3p, miR-631, miR-6071, miR-5690, miR-519e-5p, miR-519d-5p, miR-515-5p, miR-5002-3p, miR-4800-5p, miR-4755-3p, miR-4748, miR-4730, miR-4724-3p, miR-4650-3p, miR-4641, miR-4464, miR-4313, miR-4313, miR-4309, miR-4285, miR-3943, miR-376c-3p, miR-3665, miR-3665, miR-3198, miR-25-5p, miR-135b-5p, miR-135a-5p, miR-1289, miR-103b
CTNNB1	miR-892c-5p, miR-892b, miR-885-5p, miR-6837-3p, miR-6806-5p, miR-6762-3p, miR-6715b-5p, miR-660-5p, miR-6513-5p, miR-6511a-5p, miR-6506-3p, miR-624-5p, miR-6069, miR-5591-3p, miR-5586-3p, miR-526b-5p, miR-512-5p, miR-4999-5p, miR-496.1, miR-495-5p, miR-483-3p.1, miR-4776-5p, miR-4765, miR-4733-5p, miR-4708-3p, miR-4684-3p, miR-4503, miR-4255, miR-3973, miR-3691-3p, miR-3688-5p, miR-3688-5p, miR-3619-3p, miR-3162-3p, miR-3137, miR-3119, miR-214-3p, miR-1972, miR-142-3p.2, miR-1276
EDNRB	miR-7160-5p, miR-6872-3p, miR-6773-5p, miR-6759-5p, miR-6748-5p, miR-6728-5p, miR-6724-5p, miR-648, miR-588, miR-548ao-3p, miR-520e, miR-520d-3p, miR-520c-3p, miR-520b, miR-520a-3p, miR-5187-5p, miR-4746-5p, miR-4661-5p, miR-4519, miR-3945, miR-3935, miR-372-3p, miR-3688-5p, miR-367-5p, miR-3670, miR-3659, miR-3650, miR-3187-3p, miR-31-5p, miR-3156-3p, miR-302d-3p, miR-19b-3p, miR-19a-3p, miR-196b-5p, miR-196a-5p, miR-182-5p, miR-181a-3p, miR-1468-5p, miR-1294, miR-1273e
FZD4	miR-8081, miR-7704, miR-7113-3p, miR-6882-3p, miR-6826-3p, miR-6809-5p, miR-6792-3p, miR-6766-3p, miR-661, miR-5739, miR-5587-5p, miR-5581-5p, miR-4782-3p, miR-4749-3p, miR-4731-5p, miR-4646-3p, miR-449c-3p, miR-4297, miR-3938, miR-3914, miR-3166, miR-3127-5p, miR-3127-5p, miR-29c-3p, miR-29b-3p, miR-29a-3p, miR-219a-5p, miR-218-5p, miR-199b-5p, miR-199a-5p, miR-1294, miR-1251-3p, miR-124-3p.2, miR-124-3p.1, miR-124-3p.1, miR-1184, miR-101-3p.1, miR-101-3p.1, let-7g-3p, let-7a-2-3p

FZD7	miR-874-5p, miR-7515, miR-6884-5p, miR-6881-3p, miR-6878-5p, miR-6871-5p, miR-6822-3p miR-6805-5p, miR-6803-3p, miR-6788-5p, miR-6788-5p, miR-6785-3p, miR-670-5p, miR-6515-5p, miR-608, miR-532-5p, miR-485-5p, miR-4777-3p, miR-4774-3p, miR-4750-3p, miR-4725-5p, miR-4700-3p, miR-4690-5p, miR-4681, miR-4661-3p, miR-4651, miR-4633-5p, miR-4532, miR-4526, miR-451b, miR-4423-3p, miR-3937, miR-3660, miR-345-3p, miR-3162-3p, miR-30c-2-3p, miR-30c-1-3p, miR-2355-5p, miR-212-5p, miR-1281
GNAO1	miR-92b-5p, miR-7974, miR-7704, miR-7155-5p, miR-6885-5p, miR-6836-3p, miR-6829-3p, miR-6791-3p, miR-6786-5p, miR-6768-5p, miR-6742-5p, miR-6739-3p, miR-6722-5p, miR-609 miR-608, miR-550a-3p, miR-503-3p, miR-4753-3p, miR-4711-3p, miR-4651, miR-449b-5p, miR-449a, miR-4463, miR-4445-3p, miR-4288, miR-3914, miR-3675-5p, miR-34c-5p, miR-34a-5p, miR-328-5p, miR-3152-5p, miR-27a-5p, miR-200c-5p, miR-193b-3p, miR-193a-3p, miR-185-3p, miR-1307-3p, miR-1264, miR-1184, let-7d-3p
IL6	miR-760, miR-6889-3p, miR-6807-3p, miR-6787-3p, miR-676-3p, miR-664a-5p, miR-628-5p, miR-574-3p, miR-5583-5p, miR-548z, miR-548t-3p, miR-548o-3p, miR-548h-3p, miR-548d-3p, miR-548bb-3p, miR-548av-3p, miR-548ap-3p, miR-548ac, miR-548aa, miR-512-5p, miR-4795-3p, miR-4680-3p, miR-4662a-5p, miR-4641, miR-4528, miR-4500, miR-449b-3p, miR-4256, miR-3925-5p, miR-365b-3p, miR-365a-3p, miR-338-5p, miR-3138, miR-3126-3p, miR-217, miR-1-5p, miR-149-5p, miR-148a-5p, miR-1323, miR-103b
MAPK11	miR-7976, miR-760, miR-7114-3p, miR-7107-5p, miR-6889-5p, miR-6886-5p, miR-6837-5p, miR-6836-5p, miR-6789-3p, miR-6786-5p, miR-6778-5p, miR-6777-5p, miR-6752-3p, miR-6746-3p, miR-6736-3p, miR-656-5p, miR-654-5p, miR-6132, miR-5689, miR-541-3p, miR-5192, miR-516a-5p, miR-4763-5p, miR-4685-5p, miR-4645-5p, miR-4642, miR-4640-5p, miR-4640-3p, miR-4532, miR-4433b-3p, miR-4313, miR-3922-5p, miR-3684, miR-3663-3p, miR-3202, miR-296-5p, miR-1911-3p, miR-1234-3p, miR-1233-5p, miR-1180-5p
POMC	miR-7704, miR-7151-3p, miR-7111-5p, miR-6884-5p, miR-6870-5p, miR-6859-3p, miR-6852-5p, miR-6847-5p, miR-6836-5p, miR-6820-5p, miR-6813-5p, miR-6779-3p, miR-6730-5p, miR-6722-3p, miR-6134, miR-6132, miR-6087, miR-6085, miR-5698, miR-5572, miR-5095, miR-488-3p, miR-485-5p, miR-4764-5p, miR-4723-5p, miR-4692, miR-4514, miR-4438, miR-4262, miR-4257, miR-412-5p, miR-377-3p, miR-3691-5p, miR-3165, miR-1909-3p, miR-181d-5p, miR-181c-5p, miR-181b-5p, miR-181a-5p, miR-1207-5p
RAB38	miR-7161-3p, miR-7110-3p, miR-6878-5p, miR-6866-5p, miR-6833-5p, miR-6788-5p, miR-6775-3p, miR-663b, miR-650, miR-5702, miR-5690, miR-5584-5p, miR-539-3p, miR-499a-5p, miR-485-3p, miR-4802-5p, miR-4758-3p, miR-4710, miR-4666a-5p, miR-4443, miR-3612, miR-33a-3p, miR-3173-5p, miR-30e-5p, miR-30d-5p, miR-30c-5p, miR-30c-2-3p, miR-30c-1-3p, miR-30b-5p, miR-30a-5p, miR-3064-3p, miR-22-5p, miR-1972, miR-1972, miR-194-5p, miR-135a-3p, miR-1304-3p, miR-1295b-3p, miR-1291, miR-124-3p.1
SOX6	miR-96-5p, miR-942-3p, miR-93-3p, miR-8075, miR-7-5p, miR-7150, miR-7110-5p, miR-6883-5p, miR-6856-5p, miR-6825-5p, miR-6785-5p, miR-6783-5p, miR-6763-5p, miR-6755-5p, miR-6754-3p, miR-6071, miR-505-5p, miR-499b-5p, miR-4767, miR-4758-3p, miR-4725-5p, miR-4649-3p, miR-449c-5p, miR-412-3p, miR-34b-5p, miR-342-3p, miR-3157-5p, miR-3150a-3p, miR-2682-5p, miR-219a-5p, miR-219a-5p, miR-1911-5p, miR-183-5p.2, miR-183-5p.2, miR-182-5p, miR-1271-5p, miR-1249-3p, miR-107, miR-103a-3p, let-7d-5p
TYR	miR-7161-3p, miR-6851-3p, miR-6845-5p, miR-6824-5p, miR-6783-5p, miR-6775-3p, miR-6762-5p, miR-634, miR-619-3p, miR-605-3p, miR-584-3p, miR-571, miR-5586-3p, miR-552-3p, miR-490-3p, miR-4696, miR-4656, miR-4646-5p, miR-4638-3p, miR-4530, miR-450b-5p, miR-4446-3p, miR-4326, miR-4314, miR-4278, miR-3934-5p, miR-3690, miR-3650, miR-330-5p, miR-328-3p, miR-326, miR-3191-5p, miR-3179, miR-3154, miR-3120-3p, miR-27a-5p, miR-204-3p, miR-1304-3p, miR-1291, miR-1208

TYROBP	miR-96-3p, miR-939-5p, miR-889-5p, miR-7107-3p, miR-6895-3p, miR-6843-3p,miR-6842-3p, miR-6766-5p, miR-6763-5p, miR-6759-5p, miR-6756-5p, miR-6753-3p, miR-654-5p, miR-6516-3p, miR-636, miR-628-5p, miR-6090, miR-579-5p, miR-571, miR-541-3p, miR-5192, miR-4518, miR-450a-2-3p, miR-4430, miR-4417, miR-4266, miR-3977, miR-3663-5p, miR-3652, miR-3175, miR-3150a-3p, miR-27a-5p, miR-218-5p, miR-2113, miR-183-5p.2, miR-183-5p.1, miR-134-3p, miR-1343-5p, miR-1287-5p, miR-1266-5p

Supplementary Table 6. The lncRNA-miRNA-mRNA ceRNA network involved in α-MSH-induced melanogenesis.

lncRNA	miRNA	mRNA
ENST00000587528	miR-92b-5p	GNAO1
ENST00000530955	miR-892b	CTNNB1
NR_104003	miR-8081	FZD4
ENST00000606533	miR-760	IL6, MAPK11
ENST00000316807, ENST00000606533	miR-7160-5p	EDNRB
lnc-BTBD19-1:1, ENST00000606533	miR-7113-3p	FZD4
NR_040100, ENST00000316807, lnc-CYP4F22-1:1	miR-6884-5p	FZD7, POMC
ENST00000606533	miR-6883-5p	SOX6
lnc-ELAVL2-1:2	miR-6881-3p	FZD7
lnc-LNPEP-2:1	miR-6878-5p	FZD7, RAB38
lnc-LNPEP-2:1	miR-6871-5p	FZD7
lnc-TRIM37-1:2	miR-6837-3p	CTNNB1
ENST00000606533	miR-6829-3p	GNAO1
ENST00000606533, ENST00000587528	miR-6825-5p	SOX6
ENST00000414633	miR-6822-3p	FZD7
NR_040100	miR-6813-5p	POMC
ENST00000606533	miR-6810-3p	CALM2
ENST00000606533	miR-6801-3p	CALM2
ENST00000606533	miR-6791-3p	GNAO1
lnc-LNPEP-2:1	miR-6779-3p	POMC
lnc-ELAVL2-1:2	miR-6766-5p	TYROBP
ENST00000606533	miR-6763-5p	SOX6, TYROBP
NR_040100	miR-6755-5p	SOX6
lnc-RBMY1J-6:1	miR-6754-3p	SOX6
ENST00000532071	miR-663b	RAB38
lnc-CYP4F22-1:1	miR-661	FZD4
ENST00000587528	miR-654-5p	MAPK11, TYROBP
ENST00000530955	miR-6511a-5p	CTNNB1
ENST00000414633	miR-619-3p	TYR
lnc-TRIM37-1:2	miR-584-3p	TYR
lnc-ELAVL2-1:2	miR-5739	FZD4
ENST00000587528, ENST00000533322	miR-541-3p	MAPK11, TYROBP
NR_104161	miR-532-5p	FZD7

ENST00000606533	miR-5095	POMC
ENST00000414633	miR-490-3p	TYR
NR_104003	miR-4767	SOX6
lnc-TRIM37-1:2	miR-4758-3p	RAB38, SOX6
lnc-BTBD19-1:1	miR-4730	CALM2
ENST00000316807	miR-4708-3p	CTNNB1
ENST00000606533, ENST00000530955	miR-4692	POMC
ENST00000599817	miR-4656	TYR
ENST00000316807, lnc-CYP4F22-1:1	miR-4646-5p	TYR
ENST00000606533	miR-4530	TYR
NR_040100, lnc-LNPEP-2:1, ENST00000414633	miR-4518	TYROBP
ENST00000606533	miR-4430	TYROBP
lnc-RBMY1J-6:1	miR-412-3p	SOX6
ENST00000606533	miR-3943	CALM2
ENST00000530955	miR-3670	EDNRB
NR_104161	miR-3652	TYROBP
lnc-RBMY1J-6:1	miR-3612	RAB38
NR_104003, ENST00000414633	miR-34a-5p	GNAO1
ENST00000606533	miR-330-5p	TYR
NR_104003	miR-3162-3p	CTNNB1, FZD7
NR_040100	miR-3157-5p	SOX6
NR_040100	miR-3154	TYR
NR_104161	miR-3137	CTNNB1
lnc-LNPEP-2:1	miR-3127-5p	FZD4
NR_040100	miR-30c-2-3p	FZD7, RAB38
lnc-ELAVL2-1:2	miR-29a-3p	FZD4
lnc-ELAVL2-1:2	miR-2355-5p	FZD7
ENST00000606533, NR_104003	miR-214-3p	CTNNB1
ENST00000606533, ENST00000533322	miR-212-5p	FZD7
ENST00000316807	miR-181b-5p	POMC
ENST00000606533	miR-1304-3p	TYR, RAB38
ENST00000532071, ENST00000606533	miR-1291	TYR, RAB38
ENST00000606533	miR-1281	FZD7
lnc-CYP4F22-1:1	miR-1273e	EDNRB
lnc-LNPEP-2:1	miR-1266-5p	TYROBP
NR_040100, lnc-LNPEP-2:1	miR-1233-5p	MAPK11

Supplementary Table 7. The circRNA-miRNA-mRNA ceRNA network involved in α-MSH-induced melanogenesis.

circRNA	miRNA	mRNA
circ_0058630	miR-760	IL6,MAPK11
circ_0026068	miR-6885-5p	GNAO1
circ_0053692, circ_0053508	miR-6842-3p	TYROBP
circ_0050401	miR-6836-3p	GNAO1
circ_0054701	miR-6828-3p	CALM2
circ_0053508, circ_0077787	miR-6792-3p	FZD4
circ_0091223	miR-6775-3p	TYR, RAB38
circ_0020240	miR-6768-5p	GNAO1
circ_0054509	miR-6728-5p	EDNRB
circ_0083902	miR-670-5p	FZD7
circ_0073464	miR-605-3p	TYR
circ_0068584	miR-588	EDNRB
circ_0084506	miR-519e-5p	CALM2
circ_0031794, circ_0084506	miR-519d-5p	CALM2
circ_0084506	miR-515-5p	CALM2
circ_0030604	miR-483-3p.1	CTNNB1
circ_0031463	miR-4782-3p	FZD4
circ_0031728	miR-4530	TYR
circ_0067366	miR-451b	FZD7
circ_0026068, circ_0053546	miR-4326	TYR
circ_0001808	miR-4288	GNAO1
circ_0001808	miR-3977	TYROBP
circ_0053546	miR-3922-5p	MAPK11
circ_0055832	miR-3663-5p	TYROBP
circ_0026068	miR-328-5p	GNAO1, TYR
circ_0083993	miR-3191-5p	TYR
circ_0059675	miR-31-5p	EDNRB
circ_0030056	miR-3138	IL6
circ_0020240	miR-3120-3p	TYR
circ_0015211, circ_0025748	miR-22-5p	RAB38
circ_0031463	miR-219a-5p	SOX6, FZD4
circ_0074400	miR-1972	CTNNB1, RAB38
circ_0091223	miR-1291	TYR, RAB38
circ_0001808	miR-1184	FZD4, GNAO1
